# Supplementary material for: Mutations of SARS-CoV-2 nsp14 exhibit strong association with increased genome-wide mutation load
Source: PeerJ. 2020 Oct 12;8:e10181. doi: 10.7717/peerj.10181 (PMC7560320; doi:10.7717/peerj.10181)
Supplement: Supplemental Information 1 [file peerj-08-10181-s001.pdf]

Supplementary Figure 1

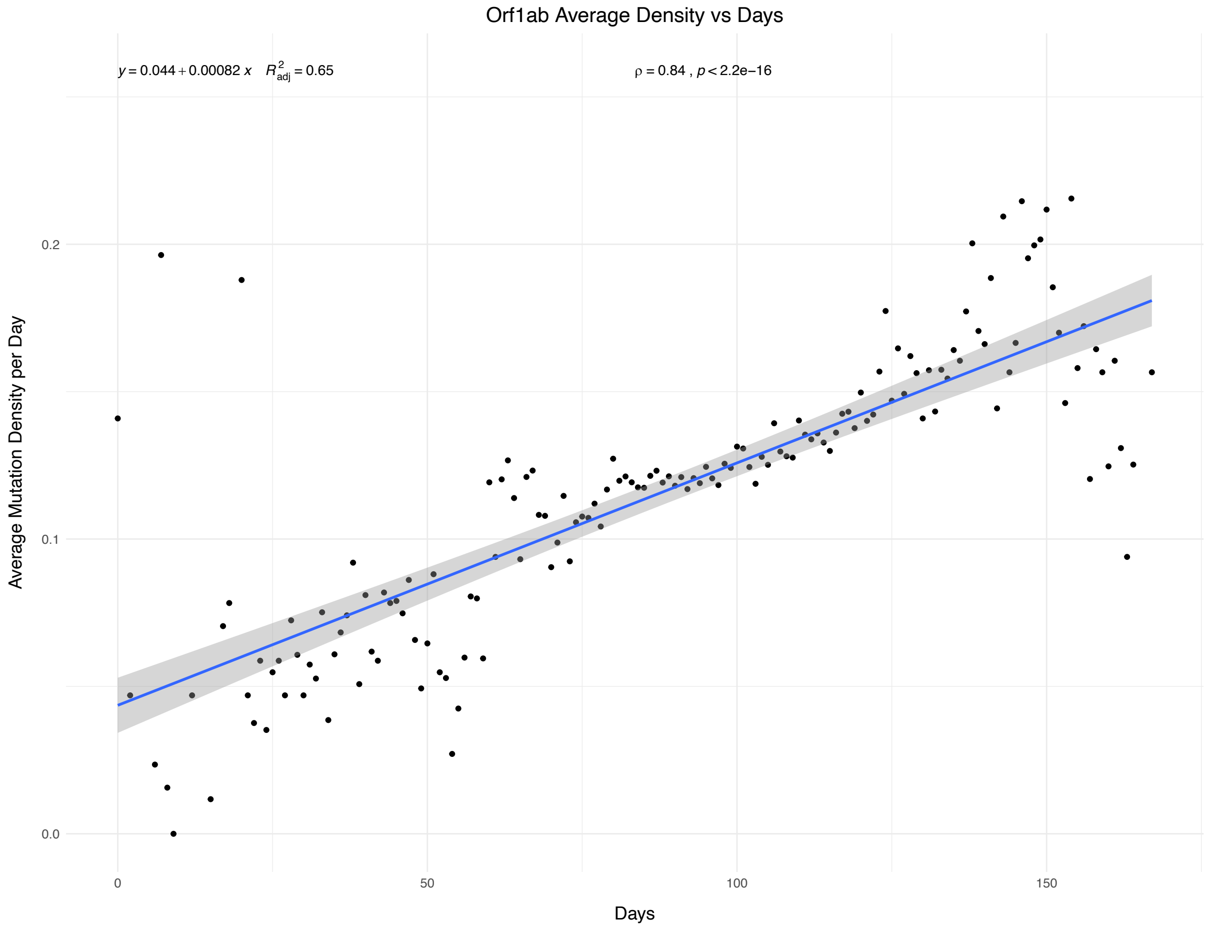

Supplementary Figure 1. The distribution of daily mutation density averages in Orf1ab gene. Correlation scores are calculated using Spearman rank correlation.

Supplementary Figure 2

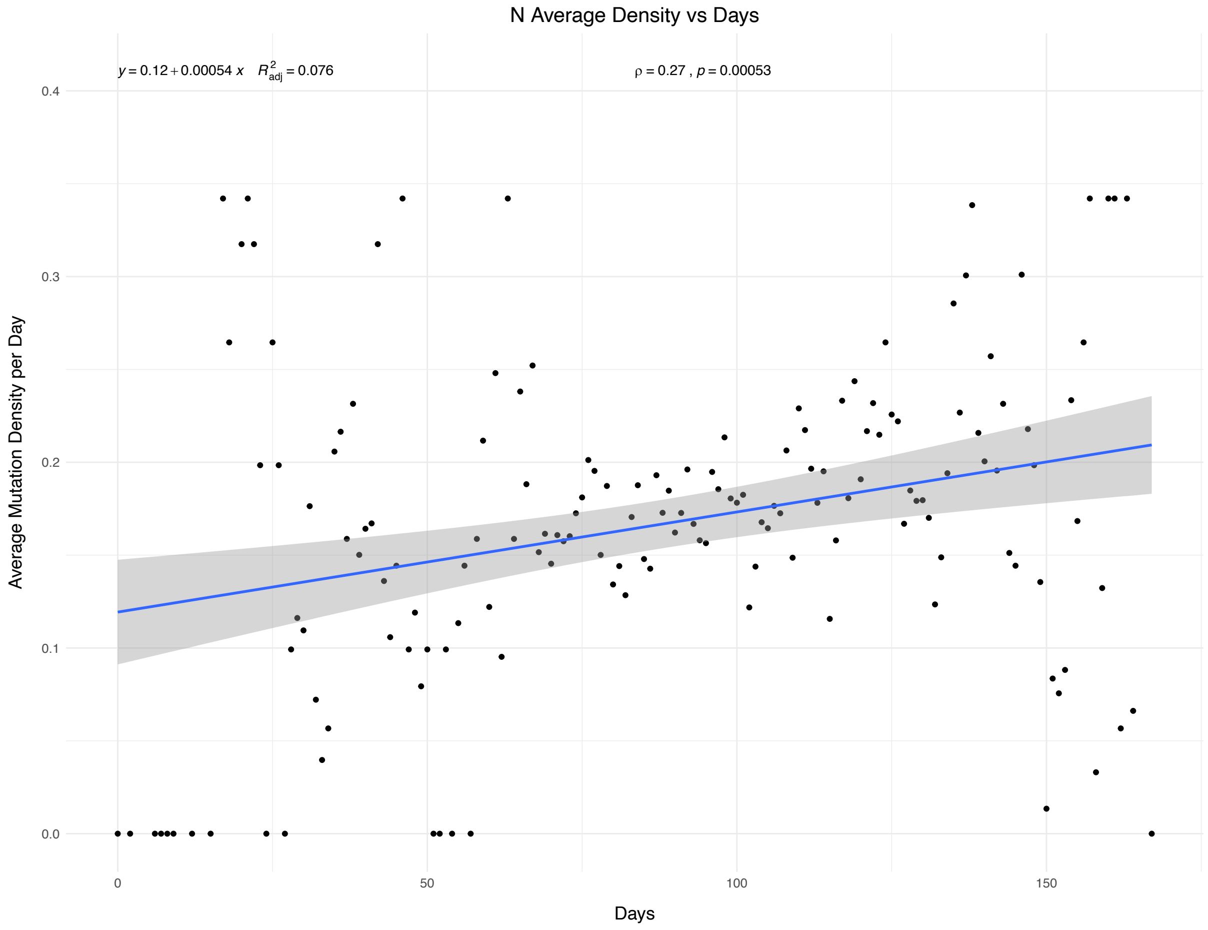

Supplementary Figure 2. The distribution of daily mutation density averages in N gene.

Correlation scores are calculated using Spearman rank correlation.

Supplementary Figure 3

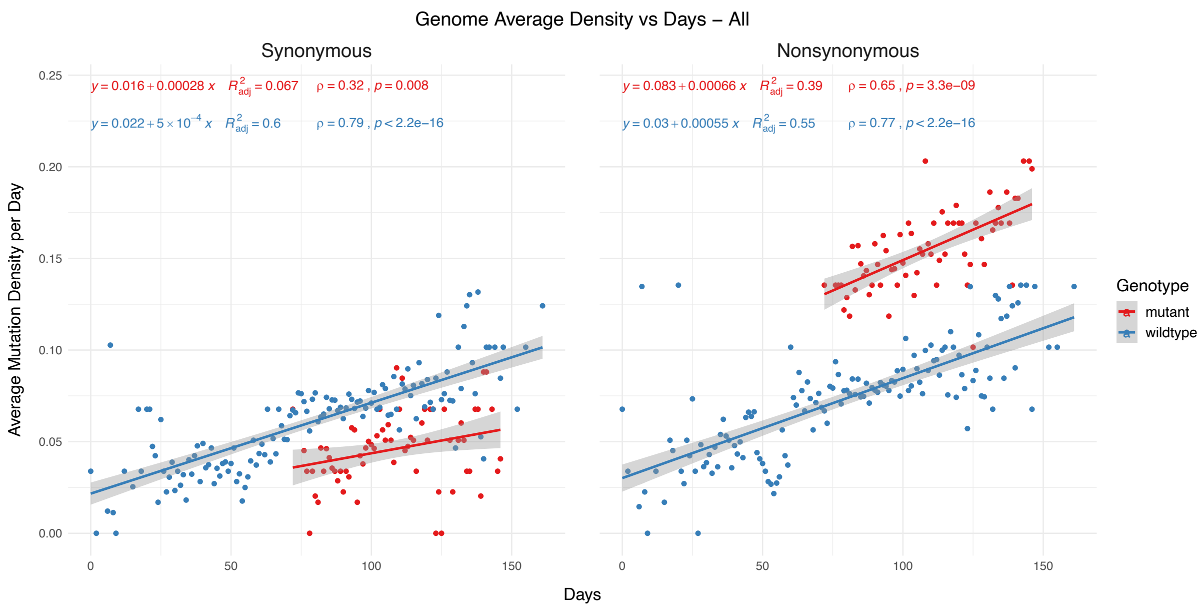

Supplementary Figure 3. The distribution of synonymous and nonsynonymous mutations in isolates carrying 11916C>T mutation compared to wildtype isolates.

Correlation scores are calculated using Spearman rank correlation.

Supplementary Figure 4

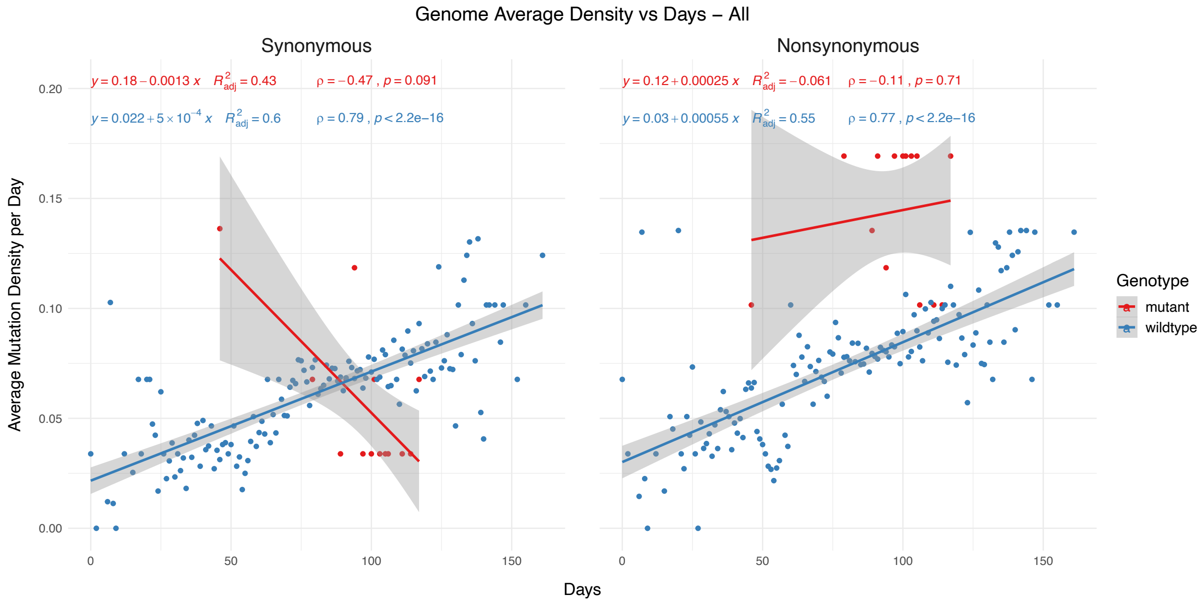

Supplementary Figure 4. The distribution of synonymous and nonsynonymous mutations in isolates carrying 12073C>T mutation compared to wildtype isolates.

Correlation scores are calculated using Spearman rank correlation.

Supplementary Figure 5

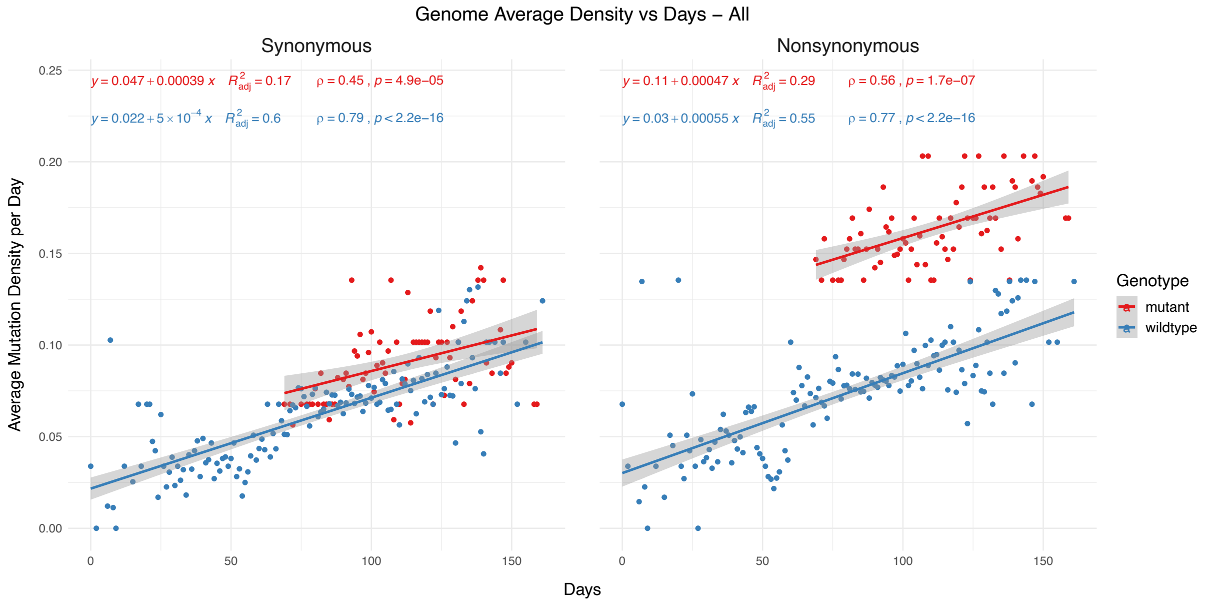

Supplementary Figure 5. The distribution of synonymous and nonsynonymous mutations in isolates carrying 13536C>T mutation compared to wildtype isolates. Correlation scores are calculated using Spearman rank correlation.

Supplementary Figure 6

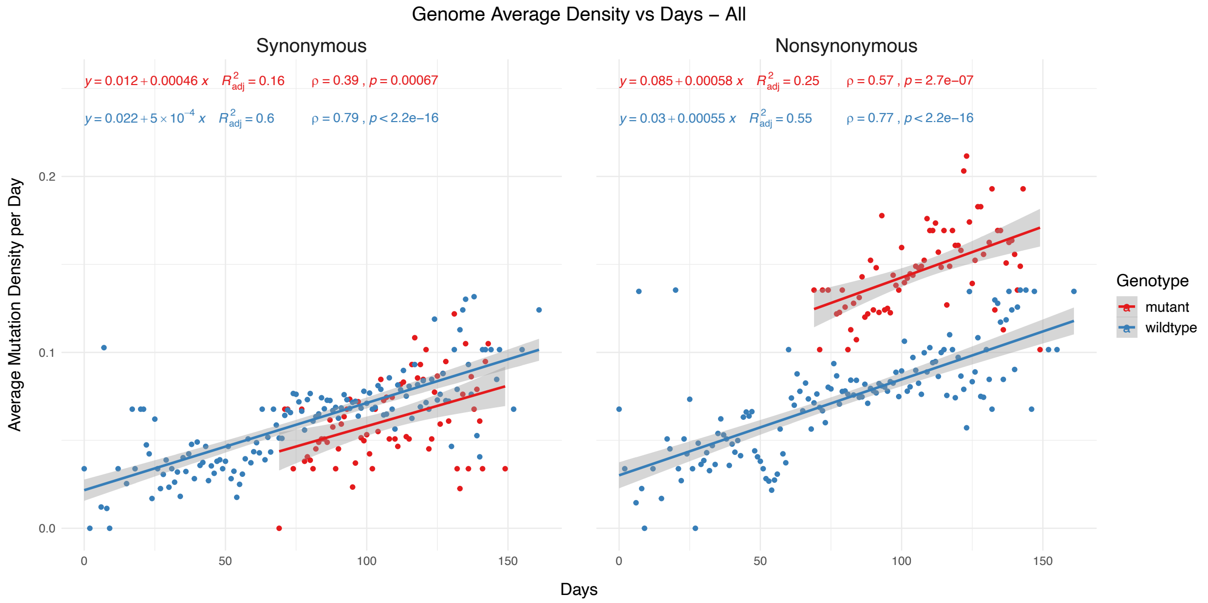

Supplementary Figure 6. The distribution of synonymous and nonsynonymous mutations in isolates carrying 13730C>T mutation compared to wildtype isolates. Correlation scores are calculated using Spearman rank correlation.

Supplementary Figure 7

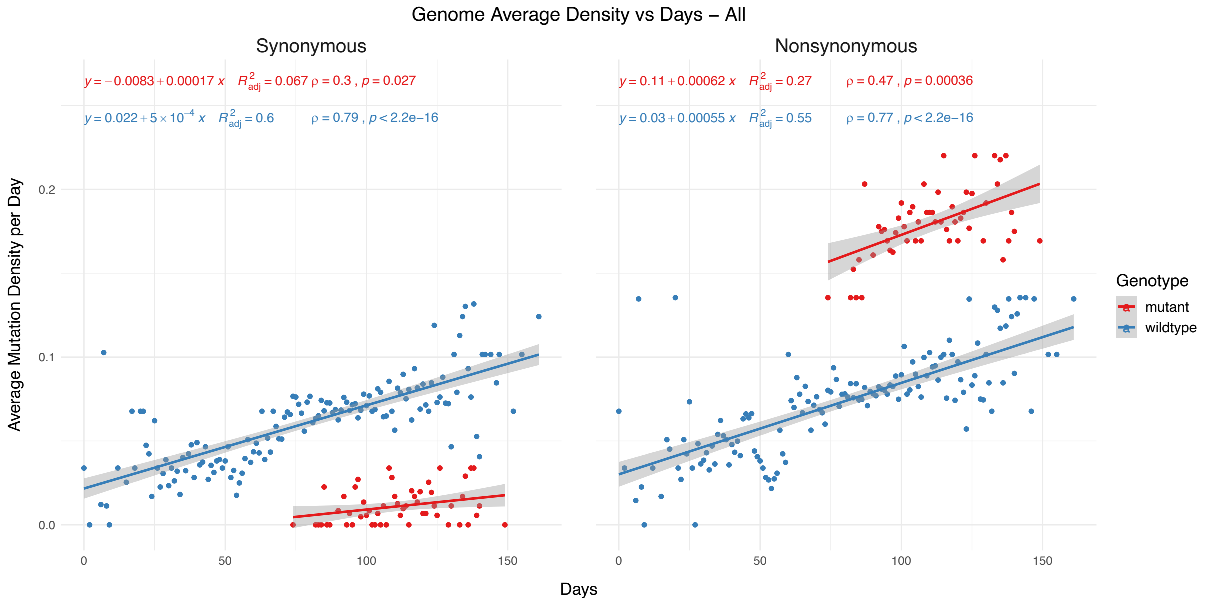

Supplementary Figure 7. The distribution of synonymous and nonsynonymous mutations in isolates carrying 13862C>T mutation compared to wildtype isolates. Correlation scores are calculated using Spearman rank correlation.

Supplementary Figure 8

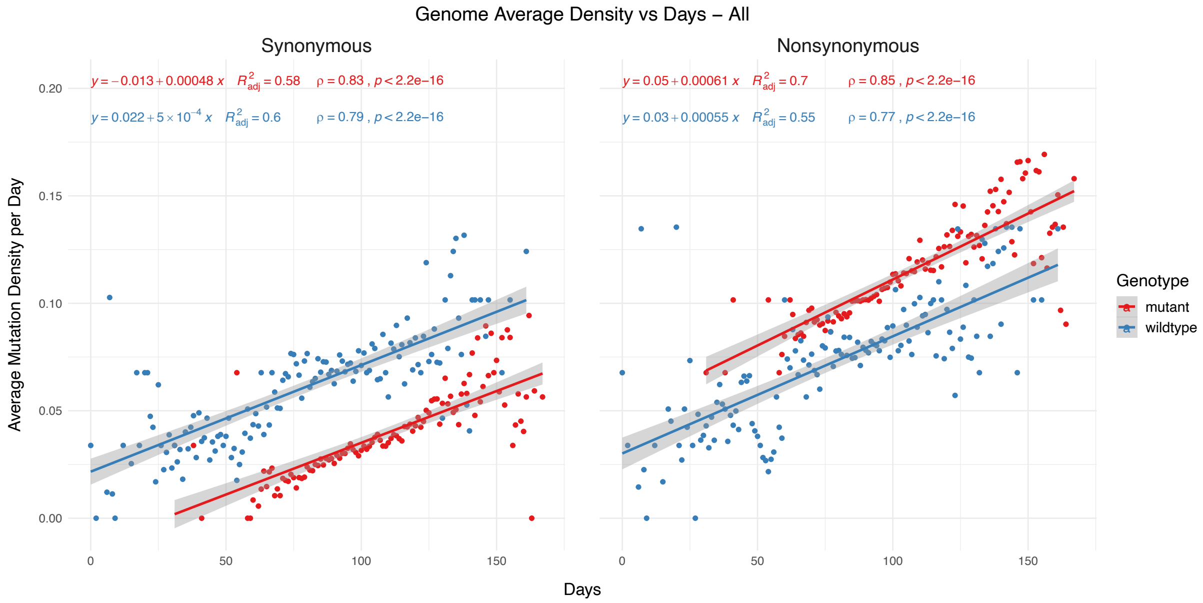

Supplementary Figure 8. The distribution of synonymous and nonsynonymous mutations in isolates carrying 14408C>T mutation compared to wildtype isolates. Correlation scores are calculated using Spearman rank correlation.
